# Retroviral matrix and lipids, the intimate interaction

**DOI:** 10.1186/1742-4690-8-15

**Published:** 2011-03-07

**Authors:** Elise Hamard-Peron, Delphine Muriaux

**Affiliations:** 1 Human Virology Department, Inserm U758, Ecole Normale Superieure de Lyon, 36 Allee d'Italie, IFR128, Universite de Lyon, Lyon, France

## Abstract

Retroviruses are enveloped viruses that assemble on the inner leaflet of cellular membranes. Improving biophysical techniques has recently unveiled many molecular aspects of the interaction between the retroviral structural protein Gag and the cellular membrane lipids. This interaction is driven by the N-terminal matrix domain of the protein, which probably undergoes important structural modifications during this process, and could induce membrane lipid distribution changes as well. This review aims at describing the molecular events occurring during MA-membrane interaction, and pointing out their consequences in terms of viral assembly. The striking conservation of the matrix membrane binding mode among retroviruses indicates that this particular step is most probably a relevant target for antiviral research.

## Introduction

Retroviruses are enveloped single-stranded RNA (+) viruses; they include some human pathogens such as human immunodeficiency virus (HIV), and oncoviruses such as the murine leukemia virus (MLV). Regardless of their diversity and the high divergence in their sequences, they share functional and viral protein structure similarities. Their genome contains the three retroviral genes: gag, pol, and env, and regulatory proteins in the case of complex retroviruses. One of the important steps in the process of retoviral infection is the formation of new infectious particles. It consists of the assembly of the viral core at the cellular membrane, budding, and maturation of the viral particles. In this review, we will focus especially on the events that occur at the molecular level during the interaction between Gag and membranes, more particularly between the Matrix domain of retroviral Gag proteins and the phospholipids, and we will place it in the context of the viral assembly process. Retroviral assembly relies on the viral Gag protein, and especially its ability to interact with the viral genomic RNA (gRNA) and cellular membranes. Gag is a polyprotein with three domains: the matrix domain, MA, that binds membranes, the capsid domain, CA, that contains Gag multimerization motifs and is responsible for the viral capsid formation (see [1] for review), and the nucleocapsid domain, NC, that recruits the RNA genome and also promotes Gag multimerization [2,3]. The assembly process most probably initiates with the formation of a ribonucleoprotein complex composed of a few Gag molecules and the gRNA, which is going to interact with membranes [4,5]. Beta-retroviruses and spumaviruses are exceptions, that fully assemble in the cytosol before reaching membranes (see [6] for review on spumaviruses, and [7] for study on the role of MA in promoting cytosolic assembly of M-PMV). The formation of higher order Gag multimers leads to the formation of the viral particle at the plasma membrane, and subsequent budding and maturation, which consist of the proteolytic cleavage of Gag and structural rearrangement of the particle. The MA domain is not only carrying Gag trafficking and membrane binding determinants, but also dictating the specificity of the bound lipid. Many data have been recently published partially unveiling the molecular mechanism of MA lipid binding, enhancing the understanding of the role played by MA during Gag membrane targeting and assembly. In the light of the literature and our experiences, this review aims at proposing biochemical models for MA-lipid interactions for different retroviruses, and replacing the consequences of such interactions in the context of retroviral assembly. We will identify the elements conserved through retroviral evolution, and those that are specific to particular retroviral strains.

## Matrix proteins: a structural point of view

**Figure 1:**
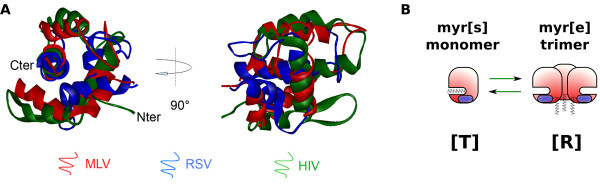
**A structural overview of retroviral MAs**. (A): Structural superimposition of MLV (1MN8), RSV (1A6S) and HIV (1TAM) MA proteins. Superimposition obtained using the combinatorial extension method (CE) and the image was generated with Viewer Pro software (Accelrys), thanks to E. Derivery. (B): Scheme of the [T] to [R] switch. [T] conformation sequesters the myristate of myristylated MAs, and remains monomeric, while [R] conformation associates in trimers and exposes the myristate (when present).

Despite low sequence similarity, MAs from different retroviruses share a conserved function in anchoring the viral Gag polyprotein to the plasma membrane. Indeed, most Gag chimeras with heterologous MA domains remain able to drive particle assembly [8-11]. One element allowing the interaction with the cellular membrane is N-terminal myristylation, a post-tranlational modification found in MAs from all retroviral families (myrMAs), including human immunodeficiency virus (HIV) [12], human T-lymphotropic virus (HTLV) [13], Mason-Pfizer monkey virus (M-PMV) [14] and exogenous murine leukemia virus (MLV) strains [15,16]. This myristate moiety is a common signal for membrane targeting of proteins, as it can insert into membrane bilayers. There are some exceptions, however, as Rous sarcoma virus (RSV), Visna virus, caprine arthritis-encephalitis virus (CAEV) and equine infectious anemia virus (EIAV) MAs are not myristylated [14]. Therefore, myristylation cannot be the only element involved in this targeting. Structural analysis of MA domains offers some clues for understanding its conserved biological role regarding membrane anchoring. Matrix structures from nine retroviruses have been resolved to date: HIV-1 [17-20] and 2 [21], SIV [22], HTLV-2 [23], bovine leukemia virus (BLV) [24], M-PMV [25], RSV [26], EIAV [27], and MLV [28]. They are all made of a globular core composed of four *α*-helices, whose overall organization is conserved among the retroviridae family [29,30] as shown by the superimposition Figure 1A. In the case of HIV-1, the unmyr-MA structure was resolved both by NMR [17,18] and crystallography [19], while the myr-MA structure was resolved by NMR only [20]. HIV-1 unmyr-MA (as well as SIV, but neither EAIV nor MLV MAs) crystallized as trimers, while it appeared mainly monomeric in classical NMR conditions. Overall structure was conserved between myr and unmyr-MA, but some differences arose, notably in the putative trimerization region and in the first alpha helix. As suggested earlier by Zhou and Resh [31], Tang and colleagues [20] showed that there is an equilibrium between two conformations of HIV-1 myrMA in solution. In the myr[s] conformation, the myristate moiety is sequestrated inside the core of the protein (see scheme in Figure 1B). This is the conformation adopted by the majority of myr-MA at a concentration of 150-200 *μ*M. The other conformation, myr[e], promotes the exposure of the myristate and tends to assemble in trimers. This conformation is probably close to the conformation observed for unmyr-MA. The conversion from one state to the other is entropically regulated [20]. In particular, high concentration of MA (more than 400 *μ*M) promotes trimerization and stabilizes the myr[e] conformation. This will be extensively discussed in the next sections. Whether these myr[s] and myr[e] conformations exist for other retroviral MAs has never been demonstrated formally. However, a NMR study carried out on EIAV-MA (which is not myristylated) evidenced amino acid shifts at high MA concentration, and correlated with an increase of the trimeric versus monomeric state [32]. Even if no major conformation change was noticed, this may correspond to an entropic switch between two slightly different conformations, similar to HIV. We, therefore, propose a new nomenclature for the MA conformations, that can also apply for unmyristylated MAs. By analogy with the enzymology, the membrane-binding prone conformation will be denoted hereafter as relaxed [R], while the other conformation will be denoted as tensed [T] (Figure 1B). Another important element of MA necessary for membrane binding is most probably the highly basic region (HBR). Indeed, an exposed patch of basic amino acids has been observed or predicted on all retroviral MAs [30]. A comparison between structurally superimposed retroviral MAs shows that this domain "migrates" on the surface of the protein, but is always found in the proximity of the N-terminus [30]. This supports the idea that the N-terminus and the polybasic region of MA cooperate for efficient membrane binding, as HBR was hypothetized to promote interaction of MA with acidic phospholipid heads [30]. Moreover, other amino acids could be involved in Gag membrane anchoring, such as the N-terminal amino acids invovled in [T] to [R] conversion in HIV-MA [33,34].

## Acidic lipid binding: the biochemical characterization

In cells, analysis confirmed that Gag membrane binding depends on this bipartite signal for most retroviruses. On one hand, the myristate moiety is, as expected, necessary to ensure membrane binding for all myristylated MAs, as shown for MLV [16,35], HIV [36], or M-PMV [37]. On the other hand, mutations in the HBR disrupted Gag membrane-binding and assembly of HIV [38-41], MLV [42,43], feline immunodeficiency virus (FIV) [44], RSV [45], HTLV-1 [46] and M-PMV [47], suggest that MA may interact with acidic membrane lipids.

**Figure 2:**
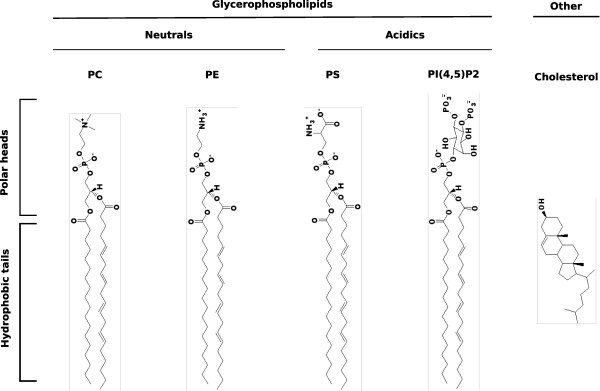
**Some lipid components of the internal leaflet of cellular membranes**. Main lipid components of the internal leaflet of cellular membranes are represented: phosphatidylcholine (PC), Phosphatidylethanolamine (PE), phosphatidylserine (PS), Phosphatidylinositol phosphates (here, PI(4,5)P_2_) and cholesterol. In membrane bilayers, the polar heads (top) face the cytosol, while the hydrophobic fatty acid chains (bottom) face the hydrophobic tails of the other leaflets's lipids.

To precisely identify the lipids that interact with retroviral Gag proteins, researchers focused on the lipids potentially present at the budding site. Phospholipids, including glycerophospholipids and sphingolipids, are the main components of cellular membranes, among which the most abundant are phosphatidylcholine (PC) and phosphatidylethanolamine (PE), both containing a neutral polar head. Some less abundant species, however, like phosphatidyl serine (PS), phosphatidyl glycerol (PG) or phosphatidylinositol phosphates (PIPs), contain acidic polar heads (cf. Figure 2). Apart from phospholipids, cellular membranes also contain other lipids, such as cholesterol, and an important proportion of transmembrane proteins. The composition of a membrane depends on its localization (internal/plasma membrane, inner/outer leaflets, etc.) and defines its functionality. Thus, retroviral assembly location restricts the panel of lipids potentially involved in the interaction with MA. Indeed, budding is mainly observed at the plasma membrane for most retroviruses, including HIV [48], M-PMV, MLV [42,49], FIV, RSV, HTLV, but may also occur on internal membranes such as endosomes (see [50] and [51] for review). Moreover, the MA domain of Gag interacts with the inner leaflet of cellular membranes, whose main lipids are PC, PE, PS, PIP (here PI(4,5)P_2_), and cholesterol [52], thereby succeptible to interact with MA (Figure 2).

Interaction between proteins and lipids can be studied i*n *vitr*o *using biomimetic membranes, and in particular large unilamellar vesicles (see [53] for review on using LUVs). The dissociation constant (Kd) can be measured, and corresponds to the lipid concentration at which half the protein is associated with the lipids: the lower the Kd, the higher the affinity. Most experiments were performed using recombinant MA proteins, because purification of the entire Gag protein is not easy. MA domain is separated from the rest of Gag by a flexible linker, thus isolated recombinant MA should recapitulate most functions of MA domain in Gag. It must be taken into account, however, that HIV-MA alone seems to have decreased affinity for membranes in comparison to the entire Gag [31]. Recombinant MA is also directly representative of the maturated MA domain function in mature particles and during early stage of viral infection. As expected, purified recombinant MAs from RSV [54] and HIV-1 [55,56] can bind containing an acidic phospholipid, the phosphatidylserine (PS), which is an abundant specy in the internal layer of cellular membranes. The order of magnitude of the Kd measurements made for recombinant RSV-MA and HIV-1 myristylated MA (myrMA) were of 10_-_^3^M, and about one order of magnitude lower upon forced dimerization of MA [54,55]. Nevertheless, the method used in these studies, i.e. LUV flotation, may underestimate the actual affinity, as the sucrose gradient may dilute the lipids. Indeed, we and others reported a value closer to 10_-_^5^M for unmyristylated HIV MA (HIV unmyrMA) by sedimentation assay [42] or by intrinsic fluorescence measurement [56,57]. Ehrlich and colleagues [56] showed that HIV-1 MA is also able to bind i*n *vitr*o *to another basic phospholipid, the phosphatidylglycerol (PG). These later studies were contested, however, because the authors also observed a binding of the CA domain of Gag to PG and PS that other authors questioned [54]. Recently, Barrera and colleagues [58] confirmed that CA has acidic lipid binding properties [58,59], rehabilitating the previous findings. It was also reported that EIAV MA can interact with PS (Kd *<*10_-_^6^M at 0.1 M NaCl) and PC [60].

The binding of retroviral MAs to lipids was thus considered to be purely electrostatic, as the interaction with PS was inhibited at high ionic strength. The Kd values found would fit well with the computational models considering electrostatic interaction between acidic lipids and basic MAs [30]. These reported Kd values would be rather low, though, to fully explain the binding of Gag to the plasma membrane in cells, and multimerization was invoked to explain MA binding to membranes [54,55].

Several retroviruses, however, show a dependency on a particular acidic phospholipid, the PI(4,5)P_2_, for efficient particle production in cells. These include HIV [61,62], M-PMV [47] and MLV [42,62]. Phosphatidylinositol phosphates are a family of acidic glycerophospholipids, with a polar head made of an inositol ring that can be mono-, bi-or tri-phosphorylated (Figure 2 shows the example of PI(4,5)P_2_). The sub-cellular localization of the different species is highly regulated by cellular kinases and phosphatases, such that they stand as major determinants of the identity of organelles' membranes(see [63], [64] and [65] for review).

The interaction between MAs and PI(4,5)P_2 _has been observed in vitro by NMR (EIAV [32], HIV-1 [66] and HIV-2 [21]), using LUVs (HIV-1 [67-69] and MLV [42]), by mass spectrometric footprinting (HIV-1 [70]) and by surface plasmon resonance (SPR)(HIV-1, [71]). The Kd values measured by NMR were rather high for all tested lentiviruses (EIAV, HIV-1, and HIV-2), ranging from 125 to 185 *μ*M, and cannot account for membrane binding in cells. It is noteworthy though that these interactions were observed with short chain PIPs (Di-C4-PI(4,5)P_2_). In contrast, SPR analysis was performed both with Di-C4-and Di-C8-PI(4,5)P_2 _(longer acyl chains), and Kd values decreased significantly in the case of Di-C8-PI(4,5)P_2_, suggesting that acyl chains are involved in the interaction between MA and PI(4,5)P_2 _[71]. The Kd of this interaction could not be calculated in the LUV systems, however, neither for the recombinant HIV MA domain [42,55], nor for the recombinant RSV MA domain [54]. This suggests that unlike PS binding, the mechanism of PIP/HIV-MA interaction could be more complex than a simple electrostatic interaction. The region of HIV-MA involved in the interaction with PI(4,5)P_2 _differs slightly depending on the method used (NMR [66] or footprinting [70]), but mapped to the HBR in both cases. New NMR techniques, using reverse micelle encapsidation instead of soluble lipids could settle it, but only preliminary results have been published to date [72].

We recently reported a definite different behavior in the case of MLV-MA [42]. UnmyrMLV-MA was able to bind PIPs-containing LUVs in a dose-dependant manner. An interaction is observed not only with PI(4,5)P_2_, but also with all the PIPs species, with Kd values ranging from 20 to 50 *μ*M. To the contrary, unmyrMLV-MA does not bind PS containing LUVs, even if the residues involved in the interaction with PIPs map to the HBR. However, adding PI(4,5)P_2 _and PS together in the same LUV dramatically increased the affinity of MLV-MA for PI(4,5)P_2_, but not for the other PIPs. Therefore, as for HIV, interaction with PIPs appears to result from a specific interaction, rather than a purely electrostatic mechanism [42].

## Specificity and regulation of the interaction with acidic phospholipids

In the light of the data presented above, we can question the specificity and the biological relevance of the interaction of retroviral Gag with the different acidic phospholipid species, as MA can interact i*n *vitr*o *with different acidic phospholipids, with important differences in Kd and interaction mode.

The lipidomics data emerging from the analysis of viral particles, however, seems to confirm the specificity for both PI(4,5)P_2 _and PS, as they are highly enriched in MLV particles [73]. This is consistent with the i*n vitro *data obtained with MLV-MA, showing that there is in fact a cooperation between PI(4,5)P_2 _and PS which allows strong MA anchoring to the membrane. Indeed, even if MLV-MA can bind any PIPs but not PS-containing LUVs, the protein actually displayed a strong stereospecificity for PI(4,5)P_2_, but exclusively when PS is added to the same LUV (resulting in a fourfold decrease in Kd, [42]). Thus, MA probably interacts with both PI(4,5)P_2 _and PS, but we hypothesize that PS binding may occur only after initial docking of MA on the PI(4,5)P_2_. In HIV particles, PI(4,5)P_2 _is enriched, while PS is present at high concentrations. Together with data emerging from MLV study, these results indicate that i*n *vitr*o *binding of HIV-MA to both PI(4,5)P_2 _and PS may be biologically relevant. Other families of lipids may also regulate MA association with membranes. In particular, HIV myrMA show more affinity for cholesterol-containing biomimetic membranes [57], and cholesterol enhances the binding specificity of HIV-MA to PI(4,5)P_2 _[67], in accordance with the finding that retroviruses can bud in cholesterol-enriched membrane domains such as lipid rafts [74-76].

**Figure 3:**
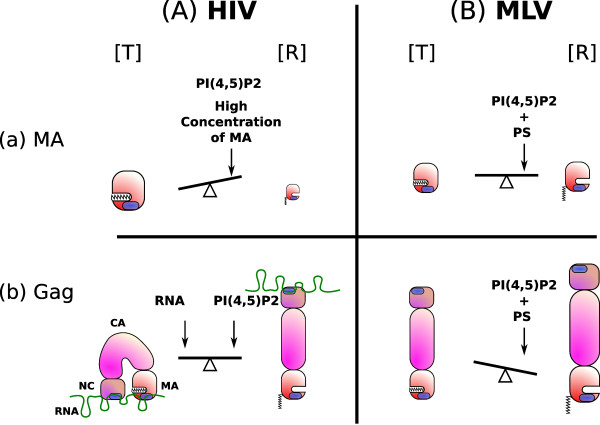
**A model for [T] to [R] equilibrium in different conditions**. Some elements are susceptible to influence the MA [T] vs [R] equilibrium, in the context of MA alone (in the mature particle, during the early step of infection, or *in vitro*), or as a domain of the Gag polyprotein. The "initial" equilibrium (in solution, purified protein, concentration around 1 *μ*M) between the [T] and [R] conformations of HIV (A) and MLV (B) MAs (a) or Gag (b) are depicted, the size of the protein representing the relative amount of each form. The factors susceptible to induce a majority of a given conformation are written in bold characters. Others, such as PI(4,5)P_2 _in the case of HIV-MA, are only able to (slightly) displace the equilibrium, even at a saturating concentration (Aa).

Surprisingly enough, another element, the RNA, was recently found to be involved in the regulation and the specificity of HIV-MA membrane binding [69]. Indeed, HIV-MA has long been known to bind RNA efficiently i*n *vitr*o *[67,70,77-79], as does BLV-MA [80] and RSV-MA [81]. Moreover, HIV-MA specifically interacts with RNA, bearing a high degree of homology to a region within the Pol open reading frame of the HIV-1 genome, suggesting that the RNA molecule interacting with MA in cells might be the viral gRNA [79]. Interestingly, the basic residues of HIV-1 MA involved in the interaction with RNA are also necessary for PI(4,5)P_2 _binding [66,70,77,79]. Thus, RNA might be a competitive inhibitor of the interaction with PI(4,5)P_2_. As a matter of fact, Chukkapalli and colleagues observed that RNAse treatment increased binding of Gag to both neutral and acidic LUVs (PC, +/- PS, +/- PI(4,5)P_2_) [69]. The hypothesis is that RNA would inhibit the entropic switch, stabilizing the [T] conformation (Figure 3Ab), thus preventing membrane-binding in general. On the other hand, Alfadhli and colleagues [67] simultaneously found that PI(4,5)P_2 _is the only lipid that can remove nucleic acids bound to HIV-1 myrMA recombinant protein. This favors the idea that RNA would ensure the specificity of the interaction of MA with the PI(4,5)P_2_, which therefore appears as a relevant cellular partner of Gag during the assembly process, allowing MA to switch from a "transport" [T] conformation to a "membrane binding" [R] conformation. RNA-meditated regulation of HIV-MA binding to PI(4,5)P_2 _seems to be supported by the data emerging from i*n *cellul*o *studies. A functional link between the genomic RNA exporting pathway and the HIV-1 MA-driven assembly has been established recently, even if the precise mechanism has not been elucidated [82-85]. Whether gRNA plays a role in MA/lipid interaction for other retroviruses is not known as yet. EIAV or MLV does not seem to have the same dependency on gRNA export pathway for proper assembly [84,85] as compared to HIV-1. In contrast, RSV-MA is able to interact with both PS [54] and RNA [81]. The measured affinity for PI(4,5)P_2 _was found to be low in the case of RSV MA alone [54], but given the results obtained with HIV-MA, further investigation could prove useful. Thus, from an evolutionary point of view, it would be interesting to determine if these regulation modes involving PI(4,5)P_2 _and RNA are conserved among retroviruses, including those lacking MA myristylation.

In summary, we have proposed a model in which two different retroviral MAs use alternative mechanisms to bind membrane lipids, but end up with the same lipid specificity. MLV-MA is able to interact initially with PI(4,5)P_2_, and this interaction triggers a conformational modification that allows PS binding. In contrast, HIV-MA would have initially low affinity for PI(4,5)P_2_, especially in the presence of gRNA [69]. However, PI(4,5)P_2 _seems to be the only compound able to compete with RNA for HIV-MA binding [67], and once RNA is removed, HIV-MA would be able to interact both with PI(4,5)P_2 _and PS, and this interaction may be stabilized by other elements, as discussed in the next section. Therefore, in spite of different lipid binding modes, the specificity of binding could be highly conserved among retroviruses.

## Let's switch again! Stabilization of the [R] conformation

**Figure 4:**
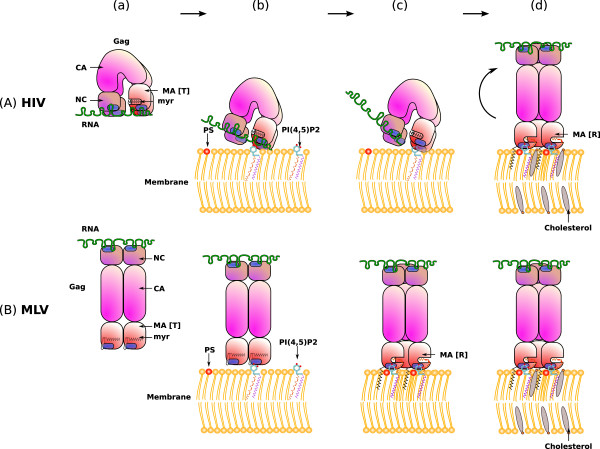
**Models for retroviral Gag membrane binding**. Aa and Ba: formation of Gag dimers, association on gRNA. Ab: inhibition of HIV-MA membrane binding by gRNA. Ac: removal of gRNA resulting from competition between gRNA and PI(4,5)P2 for HIV-MA binding. Ad: Stabilization of the [R] conformation of MA by interaction with PI(4,5)P2, Gag trimerization, stabilization of membrane anchoring by PS, lateral targeting of Gag to assembly microdomains. Bb: Binding of MLV-MA to PI(4,5)P2. Bc: Secondary binding of MLV-MA to PS, stabilization the MA [R] conformation. Bd: lateral targeting of Gag to assembly microdomains.

The interaction of retroviral MAs with PI(4,5)P_2 _seems to be a conserved, highly specific, and regulated feature among retroviruses. As previously mentioned, PI(4,5)P_2 _binding seems to be associated with conformational changes, as shown by NMR for HIV-1 MA [66] and EIAV-MA [86]. For HIV, it corresponds to the myr[s] and myr[e] conformations ([T] and [R] respectively) evidenced by structural studies [20], and it is probably also the case for EIAV except that it is not myristylated. This supports a pre-existing hypothesis first proposed by Zhou *et al *[31]: the existence of a "myristyl switch", that is actually an entropic equilibrium between the [T] conformation that sequesters the myristate inside the protein, and the [R] conformation that promotes trimeristation and exposure of the myristate moiety allowing its insertion in the cellular membranes. A refinement of this model was proposed by Saad and colleagues, as the NMR data on HIV-MA suggested that the insertion of the myristate into the lipidic bilayer may be compensated by the extraction of the 2' fatty acid chain of the PI(4,5)P_2 _out of the membrane, that would then be sequestrated into the hydrophobic core of the MA domain (Figure 4Ad) [66]. Anraku and colleagues compared the affinity of HIV-1 MA and Gag for phophorylated inositol ring alone and for medium length fatty acid chain lipids (Di-C8-PI(4,5)P_2_), in order to compare the relative contribution of electrostatic interactions (with inositol phosphate ring) and hydrophobic interactions (with acyl chains) [71]. In accordance with the data from Saad e*t *al. [66], acyl chains were found to have a major contribution in the interaction. This model, however, is built on data obtained with short chain fatty acids, and needs further confirmation in lipid bilayer conditions.

As a model for HIV-MA/PI(4,5)P_2 _interaction, we propose that the [T] conformation has a high affinity for RNA, and a low affinity for PI(4,5)P_2_. On the contrary, the [R] conformation has a high affinity for PI(4,5)P_2_. PI(4,5)P_2 _would compete with RNA for HIV-MA binding as recently proposed [69,87] and its interaction with MA would in turn stabilize the [R] conformation as shown by Saad and colleagues [66] (Figure 3Aa). In this model, PI(4,5)P_2 _has two roles: in addition to being the "substrate" (i.e. the bound molecule), it is also an effector, stabilizing the binding prone conformation, [R] (Figure 3Aa). In other words, PI(4,5)P_2 _is able to displace a pre-exiting equilibrium toward the [R] conformation, as suggested by Tang e*t *a*l *[20]. Symmetrically, RNA would have an "allosteric inhibitor" effect in stabilizing the [T] conformation (Figure 3Aa). This property may prevent a specific binding to membranes lacking PI(4,5)P_2_. This model could explain why many authors were unable to measure the affinity of HIV-1 MA for PI(4,5)P_2 _in the LUV system [42,55]. At low HIV-MA concentrations (from 1*μ*M to 20*μ*M), the equilibrium would be only slightly displaced toward the [R] conformation, even at a saturating PI(4,5)P_2 _concentration (Figure 3Aa[42,54]). The [T] conformation had very low affinity for the lipid; we and others concluded that the affinity of MA for PI(4,5)P_2 _was negligible in these conditions [42,55]. Many other elements could also influence the [T] to [R] equilibrium *in vivo*, to allow specific interaction of Gag with membranes. As mentioned earlier, a high concentration of MA promotes trimerization, and at the same time stabilizes the [R] conformation [20] (cf. Figure 3Aa). In addition, multimerization of Gag seems to correlate with the appearance of the [R] state, as multimerizing regions in CA promote myristate exposure [20] and increase lipid binding of MA-CA constructs [55]*in vitro*. In cells, it has been shown that proteolytic cleavage of Gag induces partial dissociation of p17MA from the membrane, confirming that uncleaved Gag stabilizes the [R] conformation of MA [31,88,89]. Another parameter that seems to influence the [T] to [R] transition is pH, as shown recently by Fledderman e*t *al. [90]. High pH stabilizes the [T] form, while acidification favors myristate exposure. In addition, the same laboratory also reported that Calmodulin (CalN), a Ca^2+ ^sensor protein determinant that interacts with HIV MA, promotes the myristyl switch [91].

The equilibrium constant between the [T] and [R] conformations also seems to vary greatly from one MA to another. As a matter of fact, in NMR conditions (high MA concentration, around 0.5 mM), HIV-1 and HIV-2 MAs behave differently in the presence of PI(4,5)P_2_, the [R] conformation remains undetectable for HIV-2 MA [21], unlike HIV-1 [66]. As far as other viruses are concerned, less data are available. It is possible that PI(4,5)P_2 _also stabilizes an [R] conformation of EIAV-MA as suggested by 2-D NMR data obtained by Chen *et al *[86], showing a slight amino acid shift upon PI(4,5)P_2 _binding. In contrast, MLV MA may display a more complex behavior. We were able to calculate two Kd values for MA/PI(4,5)P_2 _interaction, either in the presence or absence of PS. The [T] conformation might be able to bind PI(4,5)P_2 _with a Kd of 25 *μ*M, while the [R] conformation might be stabilized by the presence of PS, allowing PI(4,5)P_2 _to switch to the extended lipid conformation, with a resulting Kd value approaching 5 *μ*M (Figure 3Ba) [42]. Another hypothesis is that the majority of MA is already in the [R] conformation, and that PS modulates the affinity of the interaction with PI(4,5)P_2_.

The switch from the [T] to the [R] conformation may have further implications at the level of the entire Gag protein, thus influencing the assembly process. Indeed, Datta et al. recently proposed a model in which HIV-Gag would be in a bent conformation in solution, with MA and NC in close proximity [92,93] (Figure 3Ab). This model is supported by the fact that both NC and MA can bind IP6 (an inositol ring containing six phosphorylations, thus somewhat homologous to PI(4,5)P_2_) *in vitro*, and is consistent with hydrodynamic and small-angle neutron scattering data. This is also in agreement with the idea that RNA can bind both NC and MA [67,70,77-79]. This is not compatible, however, with the immature particle organization, in which Gag is in an extended rod-shaped conformation [94]. Consequently, the authors propose that viral assembly is coupled with major conformational modifications of Gag (Figure 3Ab). The same group showed that correct *in vitro *assembly of viral like particles necessitates both RNA and IP6 (that can be considered as an analog of PI(4,5)P_2_). It is still the case when the NC domain is replaced by a multimerization domain such as a leucine zipper, suggesting that RNA not only plays a role in assembly via its interaction with the NC domain, but probably also at the level of the MA domain [95].

The ability of HIV-Gag to auto-assemble into viral-like particles *in vitro *seems to be linked with a switch from Gag dimers to Gag trimers that can be mediated by IP6 [93,95]. As it has been shown that PI(4,5)P_2 _promotes HIV-MA trimeric association [87], the effect of IP6 addition could mimic the effect of PI(4,5)P_2 _binding in cells, in stabilizing the [R] conformation and promoting the formation of MA trimers. This could further trigger Gag structural reorganization via dimer to trimer transition (Figure 4Ad). A similar mechanism could drive the assembly of all retroviruses, as other retroviral MAs have multimerization properties upon PI(4,5)P_2 _binding. For exemple, MLV-MA multimerizes in the presence of PI(4,5)P_2 _under certain conditions (unpublished personal data), and EIAV-MA forms trimers [32].

MLV-Gag, however, seems to differ in some points from lentiviral Gag proteins. Datta et al. showed that in vitro recombinant MLV-Gag is readily in a rod-shaped conformation in solution, with a much more rigid structure (Datta, Zuo, Campbell, Wang, Rein: Personnal communication) (Figure 3Bb). This property might argue for an absence of an RNA mediated maintenance of the [T] conformation for MLV-MA. This correlates with the fact that the [R] conformation of MLV-MA appears more stable, as 100% of MLV-Gag is associated with membranes in cells [42], in contrast with HIV-Gag which is no more than 60% membrane bound [96]. However, we cannot exclude the possibility that RNA could regulate the interaction of MLV-MA with lipids.

The mechanisms of interaction between retroviral MAs and lipids are quite original, and whether some particularities of these binding modes can also apply to other viral or cellular proteins is not known. For instance, other retroviral proteins could interact with lipids using a similar mechanism. For example, Nef and Tat, two regulatory proteins of HIV, also bind membranes. In fact, Nef is a myristylated protein able to bind acidic phospholipids, but the curvature of the membrane induced upon Nef binding is not consistent with the extraction of a fatty acid out of the membrane [97] as in the model proposed for HIV-MA [66]. A myristyl switch mechanism is still possible, however, as the binding of Nef to biomimetic membranes is a biphasic process, with a first phase of electrostatic interaction with acidic phospholipids, and a second phase of structural modifications (in particular, the formation of an amphiphatic helix) [97]. As for Tat, it was recently shown that it also interacts with PI(4,5)P_2 _before crossing the plasma membrane and being secreted into the extracellular environment [98-100].

## Conclusion: Cellular consequence of Gag binding to PI(4,5)P_2 _and PS

Taking all the previously discussed data together allowed us to propose a model for the role played by MA during HIV and MLV assembly initiation, at the molecular level (Figure 4). In this model, Gag first polymerizes on gRNA (Aa and Ba), but adopts a bent conformation in the case of HIV (Aa), with both MA and NC interacting with gRNA, while MLV-Gag is readily in a rod-shaped conformation (Ba). For both viruses, the [T] conformation of MA is initially dominant, with myristate trapped in the protein core. When HIV-MA reaches PM (Ab), PI(4,5)P_2 _is able to compete with gRNA for MA binding (Ac). Removal of gRNA and interaction with PI(4,5)P_2 _stabilize the [R] conformation of MA (exposed myristate), which in turn promotes the trimerization and the reorganization of Gag into its rod-shapped conformation (Ad). The presence of PS could stabilize the interaction between MA and PI(4,5)P_2 _(Ad). Gag would then be laterally targeted to membrane microdomains containing high levels of saturated lipids, such as lipid rafts (Ad). In the case of MLV, initial binding to PI(4,5)P_2 _(Bb) is followed by a secondary binding to PS (Bc) that would further stabilize the [R] conformation of MA, exposing the myristate. Like HIV, lateral targeting of Gag to rafts or other microdomains is likely to occur afterwards (Bd).

These mechanistic observations are useful to re-evaluate the data available regarding assembly and budding localization in cells. Analysis of the retroviral particle envelope content evidenced that budding membranes resemble the plasma membrane in terms of lipid composition [73,75,101-105]. The ratio between lipids, however, differs from the average plasma membrane composition. In particular, viral particles of HIV and MLV are enriched not only in PI(4,5)P_2 _and PS, but also in cholesterol, ceramides, GM3 and sphingolipids [73]. This can reflect the fact that viral particles are produced in specific membrane microdomains. Moreover, HIV virions are also enriched in lipid raft markers such as GPI-anchored proteins [106], actin and actin-associated proteins, such as Ezrin-Radixin-Moesin proteins (ERMs) [107,108], and in tetraspanins [108-116]. ERM and tetraspanins are also found in particles of MLV [107,117,118]. In consequence, retroviral budding has been proposed to occur preferentially in two types of membrane microdomains associated with actin cytoskeleton: lipid rafts and tetraspanin enriched microdomains (TEMs). There is a spatial and functional distinction, however, between these two kind of domains [119-121], even if they are adjacent and may interact [122-124].

Lipid rafts are membrane domains enriched in cholesterol and sphingolipids, but can also be enriched in PI(4,5)P_2 _and PS under specific conditions [125-128]. Rafts were initially identified as detergent-resistant membranes, and this property was widely utilized to characterize raft-associated lipids and proteins, including HIV-Gag [74,129-137], MLV-Gag [76,136] and HTLV-1-Gag [136,138]. The existence in living cell, the exact nature, and the actual size of lipid rafts has, however, been intensely debated over the past decades. The current consensus is that lipid rafts are nanoscale concentrations of specific lipids, notably cholesterol and sphingolipids, and proteins (reviewed in [128,139]). Their size is around 10 to 20 nm but they can coalesce and organize membrane bioactivity in many ways.

The association of HIV-Gag with lipid rafts depends on both membrane association signals of MA, the myristate and the HBR (reviewed in [140,141]). Lower order multimerization is also necessary because the association of CA mutants with lipid rafts is delayed [74], however, higher order association appears to be dispensable as demonstrated by NC mutants [142]. Lipid raft targeting is a slower process than membrane association, giving the idea that initial docking of Gag at the plasma membrane is followed by lateral transport to assembly microdomains as proposed by Ono and Freed [74].

Saad and colleagues [66] proposed a very elegant model in agreement with a preferential budding of HIV in raft microdomains. Their NMR data suggests that the 2'-fatty acid of the PI(4,5)P_2 _is extracted from the membrane bilayer upon MA binding, and sequestrated inside the protein, in the same hydrophobic pocket the myristate occupied. Unlike the 2'-chain, the 1'-chain is usually saturated, as is the myristate (cf. Figure 4). If this model proves to be correct, Gag would then be anchored to the membrane via two saturated chains (myristate and 1'-chain) and this could result in a lateral targeting of Gag to lipid rafts, where saturated lipids are enriched (Figure 4d, Bd).

The trapping of PI(4,5)P_2 _into lipid rafts by Gag may have important consequences in terms of cellular responses. Indeed, in non-infected cells, it seems that the ratio of raft-associated PI(4,5)P_2 _versus raft-excluded PI(4,5)P_2 _is finely regulated. Any modification of one pool seems to have profound consequences, in particular on cytoskeleton remodelling, cell morphology and modulation of signaling pathways, such as the PI3K-Akt pathway [143].

Whether Gag, and in particular the MA domain, is able to aggregate lipid raft microdomains (directly or indirectly) or bind to pre-formed platforms is not as yet known, even if recent findings argue for dynamic aggregation of raft components by Gag [116]. Annexin 2 could potentially play a role, as this protein interacts with Gag [108,144] and is able to aggregate lipids, in particular cholesterol, PS, and PI(4,5)P_2 _[145,146]. Other viral proteins may be involved too. It was recently shown that gPr80^[*gag*]^, a long glycosylated form of MLV-Gag, increases the release of MLV and HIV particles via lipid rafts [76]. A similar role has been observed for HIV-Nef [147], which also increases the "raft-like" properties of HIV particles [105] and modifies the cholesterol metabolism of producer cells [148]. However, it is not known how these two proteins act to relocate assembly in these microdomains.

On the other hand, several authors have reported that retroviral assembly occurs in association with tetraspanins [108-116,149-151]. Some tetraspanins can modulate viral infectivity and regulate cell to cell transmission [115], while the role of others, such as CD63, is currently debated [152]. The tetraspanins are a family of small transmembrane proteins that operate as major lateral organizers of membrane domains. They form tetraspanin-enriched microdomains (TEMs) or tetraspanin webs, in close relation with the cytoskeleton (reviewed in [153]). TEMs are enriched in cholesterol, GM1 and sphingolipids, but only a small fraction of the tetraspanins are found in the detergent resistant membrane (DRM) fractions, unlike raft proteins. Some tetraspanins, including CD9, CD63, CD81, and CD51 are associated with PI4K, a kinase that allows the synthesis of PI(4)P, the main precurssor of PI(4,5)P_2_. In particular, HIV-Gag seems to associate specifically with CD63 and CD81 and less with CD82 [108,109,113-115] while HTLV-1 Gag associates preferentially with CD82 at the plasma membrane [149-151]. It is noteworthy that CD82 does not associate with PI4K and that this may be related to the unusual particle production mode of HTLV, with preferential budding at the cell-to-cell contact areas and low production of cell-free virions. One unresolved question is whether there is a collaboration between rafts and TEMs during particle assembly or whether distinct budding microdomains exist in the cell. In support of the first hypothesis, it was observed that some tetraspanins are able to address protein complexes toward lipid rafts, inducing the activation of specific signalization pathways. In particular, CD81 is necessary to partition the B cell receptor (BCR) and the CD19/CD21/CD81 complex into rafts [122,123], while CD82 links the actin cytoskeleton, T cell receptors and raft domains [124]. This suggests that tetraspanins may help to target Gag to lipid rafts, or, the other way around, that Gag could recruit tetraspanins and lipid raft components in order to activate particular signalization pathways necessary for sustaining HIV infection. This later model is supported by recent work by Krementsov e*t *al. showing the strong trapping of CD9 and the transient trapping of cholesterol, GM1 and CD55 into the HIV-1 assembly microdomains [116]. Interaction between TEMs and lipid rafts could result in the activation of TCR signalization pathway from which HIV could benefit. This pathway comprises, for example, the protein Lck, a Src-kinase participating in T-cell activation [154], that interacts with HIV-Gag and increases particle production [155]. Moreover, the activation of TCR not only causes the accumulation of raft lipids in the membrane areas involved in the TCR signaling pathway but also recruits PS, which is probably necessary for Gag stabilization in PM microdomains during particle formation [127].

The enriched literature on retroviral assembly has allowed us to postulate a quite precise model of the molecular events that drive the anchoring of Gag to cellular membranes preceding particle formation, but these models remain to be tested experimentally. The high conservation of the overall process is striking, especially concerning the specificity of the interaction between Matrix domain of Gag and cellular lipids (PI(4,5)P2, PS, cholesterol), and suggests that targeting retroviral assembly by therapeutical approaches may be a good strategy to combat HIV infection.

## Competing interests

The authors declare that they have no competing interests.

## Authors' contributions

EH wrote the manuscript and made the figures. DM contributed to the manuscript writing and editing. All authors read and approved the final manuscript.

## References

[B1] Adamson CS Jones IM The molecular basis of HIV capsid assembly-five years of progress Rev Med Virol 2004 14 2 107 21 10.1002/rmv.418 15027003

[B2] Darlix JL Lapadat-Tapolsky M de Rocquigny H Roques BP First glimpses at structure-function relationships of the nucleocapsid protein of retroviruses J Mol Biol 1995 254 4 523 37 10.1006/jmbi.1995.0635 7500330

[B3] Rein A Retroviral RNA packaging: a review Arch Virol Suppl 1994 9 513 22 8032280 10.1007/978-3-7091-9326-6_49

[B4] Jouvenet N Simon SM Bieniasz PD Imaging the interaction of HIV-1 genomes and Gag during assembly of individual viral particles Proc Natl Acad Sci USA 2009 106 45 19114 9 10.1073/pnas.0907364106 19861549 PMC2776408

[B5] Ott DE Coren LV Shatzer T The nucleocapsid region of human immunodeficiency virus type 1 Gag assists in the coordination of assembly and Gag processing: role for RNA-Gag binding in the early stages of assembly J Virol 2009 83 15 7718 27 10.1128/JVI.00099-09 19457986 PMC2708646

[B6] Delelis O Lehmann-Che J Saïb A Foamy viruses-a world apart Curr Opin Microbiol 2004 7 4 400 6 10.1016/j.mib.2004.06.009 15358259

[B7] Choi G Park S Choi B Hong S Lee J Hunter E Rhee SS Identification of a cytoplasmic targeting/retention signal in a retroviral Gag polyprotein J Virol 1999 73 7 5431 7 10364290 10.1128/jvi.73.7.5431-5437.1999 PMC112599

[B8] Parent LJ Wilson CB Resh MD Wills JW Evidence for a second function of the MA sequence in the Rous sarcoma virus Gag protein J Virol 1996 70 2 1016 26 http://view.ncbi.nlm.nih.gov/pubmed/8551559 8551559 10.1128/jvi.70.2.1016-1026.1996 PMC189907

[B9] Reed M Mariani R Sheppard L Pekrun K Landau NR Soong NW Chimeric human immunodeficiency virus type 1 containing murine leukemia virus matrix assembles in murine cells J Virol 2002 76 436 43 10.1128/JVI.76.1.436-443.2002 11739711 PMC135687

[B10] Chen BK Rousso I Shim S Kim PS Efficient assembly of an HIV-1/MLV Gag-chimeric virus in murine cells Proc Natl Acad Sci USA 2001 98 26 15239 44 10.1073/pnas.261563198 11742097 PMC65013

[B11] Manrique ML Gonzalez SA Affranchino JL Functional relationship between the matrix proteins of feline and simian immunodeficiency viruses Virology 2004 329 157 67 10.1016/j.virol.2004.07.029 15476883

[B12] Veronese FD Copeland TD Oroszlan S Gallo RC Sarngadharan MG Biochemical and immunological analysis of human immunodeficiency virus gag gene products p17 and p24 J Virol 1988 62 3 795 801 3123712 10.1128/jvi.62.3.795-801.1988 PMC253634

[B13] Ootsuyama Y Shimotohno K Miwa M Oroszlan S Sugimura T Myristylation of gag protein in human T-cell leukemia virus type-I and type-II Jpn J Cancer Res 1985 76 12 1132 5 3005204

[B14] Schultz AM Oroszlan S In vivo modification of retroviral gag gene-encoded polyproteins by myristic acid J Virol 1983 46 2 355 61 6302307 10.1128/jvi.46.2.355-361.1983 PMC255136

[B15] Henderson LE Krutzsch HC Oroszlan S Myristyl amino-terminal acylation of murine retrovirus proteins: an unusual post-translational proteins modification Proc Natl Acad Sci USA 1983 80 2 339 43 10.1073/pnas.80.2.339 6340098 PMC393372

[B16] Rein A McClure MR Rice NR Luftig RB Schultz AM Myristylation site in Pr65gag is essential for virus particle formation by Moloney murine leukemia virus Proc Natl Acad Sci USA 1986 83 19 7246 50 10.1073/pnas.83.19.7246 3489936 PMC386692

[B17] Massiah MA Starich MR Paschall C Summers MF Christensen AM Sundquist WI Three-dimensional structure of the human immunodeficiency virus type 1 matrix protein J Mol Biol 1994 244 2 198 223 10.1006/jmbi.1994.1719 7966331

[B18] Matthews S Barlow P Boyd J Barton G Russell R Mills H Cunningham M Meyers N Burns N Clark N Structural similarity between the p17 matrix protein of HIV-1 and interferon-gamma Nature 1994 370 6491 666 8 10.1038/370666a0 8065455

[B19] Hill CP Worthylake D Bancroft DP Christensen AM Sundquist WI Crystal structures of the trimeric human immunodeficiency virus type 1 matrix protein: implications for membrane association and assembly Proc Natl Acad Sci USA 1996 93 7 3099 104 10.1073/pnas.93.7.3099 8610175 PMC39768

[B20] Tang C Loeliger E Luncsford P Kinde I Beckett D Summers MF Entropic switch regulates myristate exposure in the HIV-1 matrix protein Proc Natl Acad Sci USA 2004 101 2 517 22 10.1073/pnas.0305665101 14699046 PMC327179

[B21] Saad JS Ablan SD Ghanam RH Kim A Andrews K Nagashima K Soheilian F Freed EO Summers MF Structure of the myristylated human immunodeficiency virus type 2 matrix protein and the role of phosphatidylinositol-(4,5)-bisphosphate in membrane targeting J Mol Biol 2008 382 2 434 47 10.1016/j.jmb.2008.07.027 18657545 PMC2581411

[B22] Rao Z Belyaev AS Fry E Roy P Jones IM Stuart DI Crystal structure of SIV matrix antigen and implications for virus assembly Nature 1995 378 6558 743 7 10.1038/378743a0 7501025

[B23] Christensen AM Massiah MA Turner BG Sundquist WI Summers MF Three-dimensional structure of the HTLV-II matrix protein and comparative analysis of matrix proteins from the different classes of pathogenic human retroviruses J Mol Biol 1996 264 5 1117 31 [Plein de refs pour trucs de base: basic residues, myr, etc HTLV-II: 4 helices alpha, une "3-10" (helice courte) patch basique]. 10.1006/jmbi.1996.0700 9000634

[B24] Matthews S Mikhailov M Burny A Roy P The solution structure of the bovine leukaemia virus matrix protein and similarity with lentiviral matrix proteins EMBO J 1996 15 13 3267 74 8670827 PMC451889

[B25] Conte MR Klikova M Hunter E Ruml T Matthews S The three-dimensional solution structure of the matrix protein from the type D retrovirus, the Mason-Pizer monkey virus, and implications for the morphology of retroviral assembly EMBO J 1997 16 19 5819 26 10.1093/emboj/16.19.5819 9312040 PMC1170213

[B26] McDonnell JM Fushman D Cahill SM Zhou W Wolven A Wilson CB Nelle TD Resh MD Wills J Cowburn D Solution structure and dynamics of the bioactive retroviral M domain from Rous sarcoma virus J Mol Biol 1998 279 4 921 8 http://view.ncbi.nlm.nih.gov/pubmed/9642071 10.1006/jmbi.1998.1788 9642071

[B27] Hatanaka H Iourin O Rao Z Fry E Kingsman A Stuart DI Structure of equine infectious anemia virus matrix protein J Virol 2002 76 4 1876 83 10.1128/JVI.76.4.1876-1883.2002 11799182 PMC135893

[B28] Riffel N Harlos K Iourin O Rao Z Kingsman A Stuart D Fry E Atomic resolution structure of Moloney murine leukemia virus matrix protein and its relationship to other retroviral matrix proteins Structure 2002 10 12 1627 36 10.1016/S0969-2126(02)00896-1 12467570

[B29] Conte MR Matthews S Retroviral matrix proteins: a structural perspective Virology 1998 246 2 191 8 10.1006/viro.1998.9206 9657938

[B30] Murray PS Li Z Wang J Tang CL Honig B Murray D Retroviral matrix domains share electrostatic homology: models for membrane binding function throughout the viral life cycle Structure 2005 13 10 1521 31 10.1016/j.str.2005.07.010 16216583

[B31] Zhou W Resh MD Differential membrane binding of the human immunodeficiency virus type 1 matrix protein J Virol 1996 70 12 8540 8 8970978 10.1128/jvi.70.12.8540-8548.1996 PMC190946

[B32] Chen K Bachtiar I Piszczek G Bouamr F Carter C Tjandra N Solution NMR characterizations of oligomerization and dynamics of equine infectious anemia virus matrix protein and its interaction with PIP2 Biochemistry 2008 47 7 1928 37 10.1021/bi701984h 18220420 PMC3120113

[B33] Paillart JC Gottlinger HG Opposing effects of human immunodeficiency virus type 1 matrix mutations support a myristyl switch model of gag membrane targeting J Virol 1999 73 4 2604 12 10074105 10.1128/jvi.73.4.2604-2612.1999 PMC104015

[B34] Saad JS Loeliger E Luncsford P Liriano M Tai J Kim A Miller J Joshi A Freed EO Summers MF Point mutations in the HIV-1 matrix protein turn off the myristyl switch J Mol Biol 2007 366 2 574 85 10.1016/j.jmb.2006.11.068 17188710 PMC1853300

[B35] Hansen M Jelinek L Whiting S Barklis E Transport and assembly of gag proteins into Moloney murine leukemia virus J Virol 1990 64 11 5306 16 1698996 10.1128/jvi.64.11.5306-5316.1990 PMC248579

[B36] Bryant M Ratner L Myristoylation-dependent replication and assembly of human immunodeficiency virus 1 Proc Natl Acad Sci USA 1990 87 2 523 7 10.1073/pnas.87.2.523 2405382 PMC53297

[B37] Rhee SS Hunter E Myristylation is required for intracellular transport but not for assembly of D-type retrovirus capsids J Virol 1987 61 4 1045 53 3493352 10.1128/jvi.61.4.1045-1053.1987 PMC254061

[B38] Yuan X Yu X Lee TH Essex M Mutations in the N-terminal region of human immunodeficiency virus type 1 matrix protein block intracellular transport of the Gag precursor J Virol 1993 67 11 6387 94 8411340 10.1128/jvi.67.11.6387-6394.1993 PMC238073

[B39] Freed EO Englund G Martin MA Role of the basic domain of human immunodeficiency virus type 1 matrix in macrophage infection J Virol 1995 69 6 3949 54 7745752 10.1128/jvi.69.6.3949-3954.1995 PMC189124

[B40] Ono A Orenstein JM Freed EO Role of the Gag matrix domain in targeting human immunodeficiency virus type 1 assembly J Virol 2000 74 6 2855 66 10.1128/JVI.74.6.2855-2866.2000 10684302 PMC111776

[B41] Zhou W Parent LJ Wills JW Resh MD Identification of a membrane-binding domain within the amino-terminal region of human immunodeficiency virus type 1 Gag protein which interacts with acidic phospholipids J Virol 1994 68 4 2556 69 8139035 10.1128/jvi.68.4.2556-2569.1994 PMC236733

[B42] Hamard-Peron E Juillard F Saad JS Roy C Roingeard P Summers MF Darlix JL Picart C Muriaux D Targeting of murine leukemia virus gag to the plasma membrane is mediated by PI(4,5)P2/PS and a polybasic region in the matrix J Virol 2010 84 503 15 10.1128/JVI.01134-09 19828619 PMC2798412

[B43] Soneoka Y Kingsman SM Kingsman AJ Mutagenesis analysis of the murine leukemia virus matrix protein: identification of regions important for membrane localization and intracellular transport J Virol 1997 71 7 5549 59 9188629 10.1128/jvi.71.7.5549-5559.1997 PMC191797

[B44] Manrique ML Celma CC Gonzalez SA Affranchino JL Mutational analysis of the feline immunodeficiency virus matrix protein Virus Res 2001 76 103 13 10.1016/S0168-1702(01)00249-0 11376850

[B45] Callahan EM Wills JW Repositioning basic residues in the M domain of the Rous sarcoma virus gag protein J Virol 2000 74 23 11222 9 10.1128/JVI.74.23.11222-11229.2000 11070020 PMC113218

[B46] Le Blanc I Rosenberg AR Dokhelar MC Multiple functions for the basic amino acids of the human T-cell leukemia virus type 1 matrix protein in viral transmission J Virol 1999 73 3 1860 7 9971764 10.1128/jvi.73.3.1860-1867.1999 PMC104426

[B47] Stansell E Apkarian R Haubova S Diehl WE Tytler EM Hunter E Basic residues in the Mason-Pfizer monkey virus gag matrix domain regulate intracellular trafficking and capsid-membrane interactions J Virol 2007 81 17 8977 88 10.1128/JVI.00657-07 17596311 PMC1951391

[B48] Hoxie JA Haggarty BS Rackowski JL Pillsbury N Levy JA Persistent noncytopathic infection of normal human T lymphocytes with AIDS-associated retrovirus Science 1985 229 4720 1400 2 10.1126/science.2994222 2994222

[B49] Suomalainen M Hultenby K Garoff H Targeting of Moloney murine leukemia virus gag precursor to the site of virus budding J Cell Biol 1996 135 6 Pt 2 1841 52 10.1083/jcb.135.6.1841 8991095 PMC2133957

[B50] Benaroch P Billard E Gaudin R Schindler M Jouve M HIV-1 assembly in macrophages Retrovirology 2010 7 29 10.1186/1742-4690-7-29 20374631 PMC2861634

[B51] Corbin A Grigorov B Roingeard P Darlix JL Muriaux D [Revisiting HIV-1 assembly] Med Sci (Paris) 2008 24 49 55 10.1051/medsci/200824149 18198110

[B52] van Meer G Voelker DR Feigenson GW Membrane lipids: where they are and how they behave Nat Rev Mol Cell Biol 2008 9 2 112 24 10.1038/nrm2330 18216768 PMC2642958

[B53] Cho W Bittova L Stahelin RV Membrane binding assays for peripheral proteins Anal Biochem 2001 296 2 153 61 10.1006/abio.2001.5225 11554709

[B54] Dalton AK Murray PS Murray D Vogt VM Biochemical characterization of rous sarcoma virus MA protein interaction with membranes J Virol 2005 79 10 6227 38 10.1128/JVI.79.10.6227-6238.2005 15858007 PMC1091718

[B55] Dalton AK Ako-Adjei D Murray PS Murray D Vogt VM Electrostatic interactions drive membrane association of the human immunodeficiency virus type 1 Gag MA domain J Virol 2007 81 12 6434 45 10.1128/JVI.02757-06 17392361 PMC1900125

[B56] Ehrlich LS Fong S Scarlata S Zybarth G Carter C Partitioning of HIV-1 Gag and Gag-related proteins to membranes Biochemistry 1996 35 3933 3943 10.1021/bi952337x 8672424

[B57] Provitera P El-Maghrabi R Scarlata S The effect of HIV-1 Gag myristoylation on membrane binding Biophys Chem 2006 119 23 32 10.1016/j.bpc.2005.08.008 16183191

[B58] Barrera FN Hurtado-Gomez E Lidon-Moya MC Neira JL Binding of the C-terminal domain of the HIV-1 capsid protein to lipid membranes: a biophysical characterization Biochem J 2006 394 Pt 1 345 53 16259620 10.1042/BJ20051487 PMC1386033

[B59] Barrera FN del Alamo M Mateu MG Neira JL Envelope lipids regulate the in vitro assembly of the HIV-1 capsid Biophys J 2008 94 2 L8 10 10.1529/biophysj.107.118083 17981892 PMC2157234

[B60] Provitera P Bouamr F Murray D Carter C Scarlata S Binding of equine infectious anemia virus matrix protein to membrane bilayers involves multiple interactions J Mol Biol 2000 296 887 898 10.1006/jmbi.1999.3482 10677289

[B61] Ono A Ablan SD Lockett SJ Nagashima K Freed EO Phosphatidylinositol (4,5) bisphosphate regulates HIV-1 Gag targeting to the plasma membrane Proc Natl Acad Sci USA 2004 101 41 14889 94 10.1073/pnas.0405596101 15465916 PMC522033

[B62] Chan WT Sherer NM Uchil PD Novak EK Swank RT Mothes W Murine leukemia virus spreading in mice impaired in the biogenesis of secretory lysosomes and Ca2+-regulated exocytosis PLoS ONE 2008 3 7 e2713 10.1371/journal.pone.0002713 18629000 PMC2443282

[B63] Di Paolo G De Camilli P Phosphoinositides in cell regulation and membrane dynamics Nature 2006 443 7112 651 7 10.1038/nature05185 17035995

[B64] Krauss M Haucke V Phosphoinositides: regulators of membrane traffic and protein function FEBS Lett 2007 581 11 2105 11 10.1016/j.febslet.2007.01.089 17316616

[B65] Krauss M Haucke V Phosphoinositide-metabolizing enzymes at the interface between membrane traffic and cell signalling EMBO Rep 2007 8 3 241 6 10.1038/sj.embor.7400919 17330069 PMC1808040

[B66] Saad JS Miller J Tai J Kim A Ghanam RH Summers MF Structural basis for targeting HIV-1 Gag proteins to the plasma membrane for virus assembly Proc Natl Acad Sci USA 2006 103 30 11364 9 10.1073/pnas.0602818103 16840558 PMC1544092

[B67] Alfadhli A Still A Barklis E Analysis of human immunodeficiency virus type 1 matrix binding to membranes and nucleic acids J Virol 2009 83 23 12196 203 10.1128/JVI.01197-09 19776118 PMC2786731

[B68] Chukkapalli V Hogue IB Boyko V Hu WS Ono A Interaction between the human immunodeficiency virus type 1 Gag matrix domain and phosphatidylinositol-(4,5)-bisphosphate is essential for efficient gag membrane binding J Virol 2008 82 5 2405 17 10.1128/JVI.01614-07 18094158 PMC2258911

[B69] Chukkapalli V Oh SJ Ono A Opposing mechanisms involving RNA and lipids regulate HIV-1 Gag membrane binding through the highly basic region of the matrix domain Proc Natl Acad Sci USA 2010 20080620 10.1073/pnas.0908661107 PMC2824378

[B70] Shkriabai N Datta SAK Zhao Z Hess S Rein A Kvaratskhelia M Interactions of HIV-1 Gag with assembly cofactors Biochemistry 2006 45 13 4077 83 10.1021/bi052308e 16566581

[B71] Anraku K Fukuda R Takamune N Misumi S Okamoto Y Otsuka M Fujita M Highly sensitive analysis of the interaction between HIV-1 Gag and phosphoinositide derivatives based on surface plasmon resonance Biochemistry 2010 49 25 5109 16 10.1021/bi9019274 20496925

[B72] Valentine KG Peterson RW Saad JS Summers MF Xu X Ames JB Wand AJ Reverse Micelle Encapsulation of Membrane-Anchored Proteins for Solution NMR Studies Structure 2010 18 9 16 10.1016/j.str.2009.11.010 20152148 PMC2876244

[B73] Chan R Uchil PD Jin J Shui G Ott DE Mothes W Wenk MR Retroviruses human immunodeficiency virus and murine leukemia virus are enriched in phosphoinositides J Virol 2008 82 22 11228 38 http://view.ncbi.nlm.nih.gov/pubmed/18799574 10.1128/JVI.00981-08 18799574 PMC2573248

[B74] Ono A Freed EO Plasma membrane rafts play a critical role in HIV-1 assembly and release Proc Natl Acad Sci USA 2001 98 24 13925 30 10.1073/pnas.241320298 11717449 PMC61143

[B75] Brugger B Glass B Haberkant P Leibrecht I Wieland FT Krausslich HG The HIV lipidome: a raft with an unusual composition Proc Natl Acad Sci USA 2006 103 8 2641 6 10.1073/pnas.0511136103 16481622 PMC1413831

[B76] Nitta T Kuznetsov Y McPherson A Fan H Murine leukemia virus glycosylated Gag (gPr80gag) facilitates interferon-sensitive virus release through lipid rafts Proc Natl Acad Sci USA 2010 107 3 1190 5 10.1073/pnas.0908660107 20080538 PMC2824291

[B77] Cimarelli A Luban J Translation elongation factor 1-alpha interacts specifically with the human immunodeficiency virus type 1 Gag polyprotein J Virol 1999 73 7 5388 401 [Montre que MA interagit avec facteur+ARN]. 10364286 10.1128/jvi.73.7.5388-5401.1999 PMC112595

[B78] Lochrie MA Waugh S Pratt DGJ Clever J Parslow TG Polisky B In vitro selection of RNAs that bind to the human immunodeficiency virus type-1 gag polyprotein Nucleic Acids Res 1997 25 14 2902 10 10.1093/nar/25.14.2902 9207041 PMC146801

[B79] Purohit P Dupont S Stevenson M Green MR Sequence-specific interaction between HIV-1 matrix protein and viral genomic RNA revealed by in vitro genetic selection RNA 2001 7 4 576 84 10.1017/S1355838201002023 11345436 PMC1370111

[B80] Wang H Norris KM Mansky LM Involvement of the matrix and nucleocapsid domains of the bovine leukemia virus Gag polyprotein precursor in viral RNA packaging J Virol 2003 77 17 9431 8 10.1128/JVI.77.17.9431-9438.2003 12915558 PMC187409

[B81] Parent LJ Cairns TM Albert JA Wilson CB Wills JW Craven RC RNA dimerization defect in a Rous sarcoma virus matrix mutant J Virol 2000 74 164 72 10.1128/JVI.74.1.164-172.2000 10590103 PMC111525

[B82] Sherer NM Swanson CM Papaioannou S Malim MH Matrix mediates the functional link between human immunodeficiency virus type 1 RNA nuclear export elements and the assembly competency of Gag in murine cells J Virol 2009 83 17 8525 35 10.1128/JVI.00699-09 19535446 PMC2738188

[B83] Hubner W Chen BK Inhibition of viral assembly in murine cells by HIV-1 matrix Virology 2006 352 27 38 10.1016/j.virol.2006.04.024 16750235

[B84] Jin J Sturgeon T Chen C Watkins SC Weisz OA Montelaro RC Distinct intracellular trafficking of equine infectious anemia virus and human immunodeficiency virus type 1 Gag during viral assembly and budding revealed by bimolecular fluorescence complementation assays J Virol 2007 81 20 11226 35 10.1128/JVI.00431-07 17686839 PMC2045577

[B85] Jin J Sturgeon T Weisz OA Mothes W Montelaro RC HIV-1 matrix dependent membrane targeting is regulated by Gag mRNA tracking PLoS One 2009 4 8 e6551 10.1371/journal.pone.0006551 19662089 PMC2717210

[B86] Chen C Jin J Rubin M Huang L Sturgeon T Weixel KM Stolz DB Watkins SC Bamburg JR Weisz OA Montelaro RC Association of gag multimers with filamentous actin during equine infectious anemia virus assembly Curr HIV Res 2007 5 3 315 23 10.2174/157016207780636542 17504173

[B87] Alfadhli A Barklis RL Barklis E HIV-1 matrix organizes as a hexamer of trimers on membranes containing phosphatidylinositol-(4,5)-bisphosphate Virology 2009 387 2 466 72 10.1016/j.virol.2009.02.048 19327811 PMC2680355

[B88] Hermida-Matsumoto L Resh MD Human immunodeficiency virus type 1 protease triggers a myristoyl switch that modulates membrane binding of Pr55(gag) and p17MA J Virol 1999 73 3 1902 8 9971769 10.1128/jvi.73.3.1902-1908.1999 PMC104431

[B89] Resh MD A myristoyl switch regulates membrane binding of HIV-1 Gag Proc Natl Acad Sci USA 2004 101 2 417 8 10.1073/pnas.0308043101 14707265 PMC327161

[B90] Fledderman EL Fujii K Ghanam RH Waki K Prevelige PE Freed EO Saad JS Myristate Exposure in the Human Immunodeficiency Virus Type 1 Matrix Protein Is Modulated by pH Biochemistry 2010 20886905 10.1021/bi101245j PMC3032006

[B91] Ghanam RH Fernandez TF Fledderman EL Saad JS Binding of calmodulin to the HIV-1 matrix protein triggers myristate exposure J Biol Chem 2010 20956522 10.1074/jbc.M110.179093 PMC3009918

[B92] Datta SAK Curtis JE Ratcliff W Clark PK Crist RM Lebowitz J Krueger S Rein A Conformation of the HIV-1 Gag protein in solution J Mol Biol 2007 365 3 812 24 10.1016/j.jmb.2006.10.073 17097677 PMC1866279

[B93] Datta SAK Zhao Z Clark PK Tarasov S Alexandratos JN Campbell SJ Kvaratskhelia M Lebowitz J Rein A Interactions between HIV-1 Gag molecules in solution: an inositol phosphate-mediated switch J Mol Biol 2007 365 3 799 811 10.1016/j.jmb.2006.10.072 17098251 PMC1829305

[B94] Ako-Adjei D Johnson MC Vogt VM The retroviral capsid domain dictates virion size, morphology, and coassembly of gag into virus-like particles J Virol 2005 79 21 13463 72 10.1128/JVI.79.21.13463-13472.2005 16227267 PMC1262573

[B95] Crist RM Datta SAK Stephen AG Soheilian F Mirro J Fisher RJ Nagashima K Rein A Assembly properties of human immunodeficiency virus type 1 Gag-leucine zipper chimeras: implications for retrovirus assembly J Virol 2009 83 5 2216 25 10.1128/JVI.02031-08 19073719 PMC2643709

[B96] Ono A Freed EO Binding of human immunodeficiency virus type 1 Gag to membrane: role of the matrix amino terminus J Virol 1999 73 5 4136 44 10196310 10.1128/jvi.73.5.4136-4144.1999 PMC104193

[B97] Gerlach H Laumann V Martens S Becker CFW Goody RS Geyer M HIV-1 Nef membrane association depends on charge, curvature, composition and sequence Nat Chem Biol 2010 6 46 53 10.1038/nchembio.268 19935658

[B98] Yezid H Konate K Debaisieux S Bonhoure A Beaumelle B Mechanism for HIV-1 Tat insertion into the endosome membrane J Biol Chem 2009 284 34 22736 46 10.1074/jbc.M109.023705 19549783 PMC2755682

[B99] Rayne F Debaisieux S Bonhoure A Beaumelle B HIV-1 Tat is unconventionally secreted through the plasma membrane Cell Biol Int 2010 34 4 409 13 10.1042/CBI20090376 19995346

[B100] Rayne F Debaisieux S Yezid H Lin YL Mettling C Konate K Chazal N Arold ST Pugniere M Sanchez F Bonhoure A Briant L Loret E Roy C Beaumelle B Phosphatidylinositol-(4,5)-bisphosphate enables efficient secretion of HIV-1 Tat by infected T-cells EMBO J 2010 29 8 1348 62 10.1038/emboj.2010.32 20224549 PMC2868572

[B101] Richieri SP Bartholomew R Aloia RC Savary J Gore R Holt J Ferre F Musil R Tian HR Trauger R Lowry P Jensen F Carlo DJ Maigetter RZ Prior CP Characterization of highly purified, inactivated HIV-1 particles isolated by anion exchange chromatography Vaccine 1998 16 2-3 119 29 10.1016/S0264-410X(97)00196-5 9607019

[B102] Aloia RC Jensen FC Curtain CC Mobley PW Gordon LM Lipid composition and fluidity of the human immunodeficiency virus Proc Natl Acad Sci USA 1988 85 3 900 4 10.1073/pnas.85.3.900 2829209 PMC279664

[B103] Aloia RC Tian H Jensen FC Lipid composition and fluidity of the human immunodeficiency virus envelope and host cell plasma membranes Proc Natl Acad Sci USA 1993 90 11 5181 5 10.1073/pnas.90.11.5181 8389472 PMC46679

[B104] Lorizate M Brugger B Akiyama H Glass B Muller B Anderluh G Wieland FT Kra¨usslich HG Probing HIV-1 membrane liquid order by Laurdan staining reveals producer cell-dependent differences J Biol Chem 2009 284 33 22238 47 10.1074/jbc.M109.029256 19553682 PMC2755948

[B105] Brugger B Krautkra¨mer E Tibroni N Munte CE Rauch S Leibrecht I Glass B Breuer S Geyer M Krausslich HG Kalbitzer HR Wieland FT Fackler OT Human immunodeficiency virus type 1 Nef protein modulates the lipid composition of virions and host cell membrane microdomains Retrovirology 2007 4 70 10.1186/1742-4690-4-70 17908312 PMC2065869

[B106] Saifuddin M Parker CJ Peeples ME Gorny MK Zolla-Pazner S Ghassemi M Rooney IA Atkinson JP Spear GT Role of virion-associated glycosylphosphatidylinositol-linked proteins CD55 and CD59 in complement resistance of cell line-derived and primary isolates of HIV-1 J Exp Med 1995 182 2 501 9 10.1084/jem.182.2.501 7543140 PMC2192116

[B107] Ott DE Coren LV Kane BP Busch LK Johnson DG Sowder RCn Chertova EN Arthur LO Henderson LE Cytoskeletal proteins inside human immunodeficiency virus type 1 virions J Virol 1996 70 11 7734 43 8892894 10.1128/jvi.70.11.7734-7743.1996 PMC190843

[B108] Chertova E Chertov O Coren LV Roser JD Trubey CM Bess JWJ Sowder RCn Barsov E Hood BL Fisher RJ Nagashima K Conrads TP Veenstra TD Lifson JD Ott DE Proteomic and biochemical analysis of purified human immunodeficiency virus type 1 produced from infected monocyte-derived macrophages J Virol 2006 80 18 9039 52 10.1128/JVI.01013-06 16940516 PMC1563931

[B109] Orentas RJ Hildreth JE Association of host cell surface adhesion receptors and other membrane proteins with HIV and SIV AIDS Res Hum Retroviruses 1993 9 11 1157 65 10.1089/aid.1993.9.1157 8312057

[B110] Jolly C Sattentau QJ Human immunodeficiency virus type 1 assembly, budding, and cell-cell spread in T cells take place in tetraspanin-enriched plasma membrane domains J Virol 2007 81 15 7873 84 10.1128/JVI.01845-06 17522207 PMC1951303

[B111] Nydegger S Khurana S Krementsov DN Foti M Thali M Mapping of tetraspanin-enriched microdomains that can function as gateways for HIV-1 J Cell Biol 2006 173 5 795 807 10.1083/jcb.200508165 16735575 PMC2063894

[B112] Sato K Aoki J Misawa N Daikoku E Sano K Tanaka Y Koyanagi Y Modulation of human immunodeficiency virus type 1 infectivity through incorporation of tetraspanin proteins J Virol 2008 82 2 1021 33 10.1128/JVI.01044-07 17989173 PMC2224585

[B113] Grigorov B Attuil-Audenis V Perugi F Nedelec M Watson S Pique C Darlix JL Conjeaud H Muriaux D A role for CD81 on the late steps of HIV-1 replication in a chronically infected T cell line Retrovirology 2009 6 28 10.1186/1742-4690-6-28 19284574 PMC2657109

[B114] Krementsov DN Weng J Lambele M Roy NH Thali M Tetraspanins regulate cell-to-cell transmission of HIV-1 Retrovirology 2009 6 64 10.1186/1742-4690-6-64 19602278 PMC2714829

[B115] Thali M The roles of tetraspanins in HIV-1 replication Curr Top Microbiol Immunol 2009 339 85 102 full_text 20012525 10.1007/978-3-642-02175-6_5 PMC4067973

[B116] Krementsov DN Rassam P Margeat E Roy NH Schneider-Schaulies J Milhiet PE Thali M HIV-1 assembly differentially alters dynamics and partitioning of tetraspanins and raft components Traffic 2010 20727121 10.1111/j.1600-0854.2010.01111.x PMC4073295

[B117] Nermut MV Wallengren K Pager J Localization of actin in Moloney murine leukemia virus by immunoelectron microscopy Virology 1999 260 23 34 10.1006/viro.1999.9803 10405353

[B118] Ott DE Coren LV Johnson DG Kane BP Sowder RCn Kim YD Fisher RJ Zhou XZ Lu KP Henderson LE Actin-binding cellular proteins inside human immunodeficiency virus type 1 Virology 2000 266 42 51 10.1006/viro.1999.0075 10612659

[B119] Espenel C Margeat E Dosset P Arduise C Le Grimellec C Royer CA Boucheix C Rubinstein E Milhiet PE Single-molecule analysis of CD9 dynamics and partitioning reveals multiple modes of interaction in the tetraspanin web J Cell Biol 2008 182 4 765 76 10.1083/jcb.200803010 18710926 PMC2518714

[B120] Wright MD Moseley GW van Spriel AB Tetraspanin microdomains in immune cell signalling and malignant disease Tissue Antigens 2004 64 5 533 42 10.1111/j.1399-0039.2004.00321.x 15496196

[B121] Yang X Kovalenko OV Tang W Claas C Stipp CS Hemler ME Palmitoylation supports assembly and function of integrin-tetraspanin complexes J Cell Biol 2004 167 6 1231 40 10.1083/jcb.200404100 15611341 PMC2172609

[B122] Cherukuri A Shoham T Sohn HW Levy S Brooks S Carter R Pierce SK The tetraspanin CD81 is necessary for partitioning of coligated CD19/CD21-B cell antigen receptor complexes into signaling-active lipid rafts J Immunol 2004 172 370 80 14688345 10.4049/jimmunol.172.1.370

[B123] Cherukuri A Carter RH Brooks S Bornmann W Finn R Dowd CS Pierce SK B cell signaling is regulated by induced palmitoylation of CD81 J Biol Chem 2004 279 30 31973 82 10.1074/jbc.M404410200 15161911

[B124] Delaguillaumie A Harriague J Kohanna S Bismuth G Rubinstein E Seigneuret M Conjeaud H Tetraspanin CD82 controls the association of cholesterol-dependent microdomains with the actin cytoskeleton in T lymphocytes: relevance to co-stimulation J Cell Sci 2004 117 Pt 22 5269 82 10.1242/jcs.01380 15454569

[B125] Munro S Lipid rafts: elusive or illusive? Cell 2003 115 4 377 88 10.1016/S0092-8674(03)00882-1 14622593

[B126] Simons K Vaz WLC Model systems, lipid rafts, and cell membranes Annu Rev Biophys Biomol Struct 2004 33 269 95 10.1146/annurev.biophys.32.110601.141803 15139814

[B127] Zech T Ejsing CS Gaus K de Wet B Shevchenko A Simons K Harder T Accumulation of raft lipids in T-cell plasma membrane domains engaged in TCR signalling EMBO J 2009 28 5 466 76 10.1038/emboj.2009.6 19177148 PMC2657588

[B128] Lingwood D Simons K Lipid rafts as a membrane-organizing principle Science 2010 327 5961 46 50 10.1126/science.1174621 20044567

[B129] Campbell S Gaus K Bittman R Jessup W Crowe S Mak J The raft-promoting property of virion-associated cholesterol, but not the presence of virion-associated Brij 98 rafts, is a determinant of human immunodeficiency virus type 1 infectivity J Virol 2004 78 19 10556 65 10.1128/JVI.78.19.10556-10565.2004 15367622 PMC516414

[B130] Ding L Derdowski A Wang JJ Spearman P Independent segregation of human immunodeficiency virus type 1 Gag protein complexes and lipid rafts J Virol 2003 77 3 1916 26 10.1128/JVI.77.3.1916-1926.2003 12525626 PMC140875

[B131] Halwani R Khorchid A Cen S Kleiman L Rapid localization of Gag/GagPol complexes to detergent-resistant membrane during the assembly of human immunodeficiency virus type 1 J Virol 2003 77 7 3973 84 10.1128/JVI.77.7.3973-3984.2003 12634357 PMC150626

[B132] Holm K Weclewicz K Hewson R Suomalainen M Human immunodeficiency virus type 1 assembly and lipid rafts: Pr55(gag) associates with membrane domains that are largely resistant to Brij98 but sensitive to Triton X-100 J Virol 2003 77 8 4805 17 10.1128/JVI.77.8.4805-4817.2003 12663787 PMC152122

[B133] Lindwasser OW Resh MD Multimerization of human immunodeficiency virus type 1 Gag promotes its localization to barges, raft-like membrane microdomains J Virol 2001 75 17 7913 24 10.1128/JVI.75.17.7913-7924.2001 11483736 PMC115035

[B134] Nguyen DH Hildreth JE Evidence for budding of human immunodeficiency virus type 1 selectively from glycolipid-enriched membrane lipid rafts J Virol 2000 74 7 3264 72 10.1128/JVI.74.7.3264-3272.2000 10708443 PMC111827

[B135] Ono A Waheed AA Freed EO Depletion of cellular cholesterol inhibits membrane binding and higher-order multimerization of human immunodeficiency virus type 1 Gag Virology 2007 360 27 35 10.1016/j.virol.2006.10.011 17095032 PMC1945131

[B136] Pickl WF Pimentel-Mui¨nos FX Seed B Lipid rafts and pseudotyping J Virol 2001 75 15 7175 83 10.1128/JVI.75.15.7175-7183.2001 11435598 PMC114446

[B137] Bhattacharya J Repik A Clapham PR Gag regulates association of human immunodeficiency virus type 1 envelope with detergent-resistant membranes J Virol 2006 80 11 5292 300 10.1128/JVI.01469-05 16699009 PMC1472128

[B138] Feng X Heyden NV Ratner L Alpha interferon inhibits human T-cell leukemia virus type 1 assembly by preventing Gag interaction with rafts J Virol 2003 77 24 13389 95 10.1128/JVI.77.24.13389-13395.2003 14645593 PMC296084

[B139] Lingwood D Kaiser HJ Levental I Simons K Lipid rafts as functional heterogeneity in cell membranes Biochem Soc Trans 2009 37 Pt 5 955 60 10.1042/BST0370955 19754431

[B140] Ono A HIV-1 Assembly at the Plasma Membrane: Gag Trafficking and Localization Future Virol 2009 4 3 241 257 10.2217/fvl.09.4 19802344 PMC2676728

[B141] Waheed AA Freed EO Lipids and membrane microdomains in HIV-1 replication Virus Res 2009 143 2 162 76 10.1016/j.virusres.2009.04.007 19383519 PMC2731011

[B142] Ono A Waheed AA Joshi A Freed EO Association of human immunodeficiency virus type 1 gag with membrane does not require highly basic sequences in the nucleocapsid: use of a novel Gag multimerization assay J Virol 2005 79 22 14131 40 10.1128/JVI.79.22.14131-14140.2005 16254348 PMC1280195

[B143] Johnson CM Chichili GR Rodgers W Compartmentalization of phosphatidylinositol 4,5-bisphosphate signaling evidenced using targeted phosphatases J Biol Chem 2008 283 44 29920 8 10.1074/jbc.M805921200 18723502 PMC2573053

[B144] Harrist AV Ryzhova EV Harvey T Gonzalez-Scarano F Anx2 interacts with HIV-1 Gag at phosphatidylinositol (4,5) bisphosphate-containing lipid rafts and increases viral production in 293T cells PLoS One 2009 4 3 e5020 10.1371/journal.pone.0005020 19325895 PMC2657825

[B145] Chasserot-Golaz S Vitale N Umbrecht-Jenck E Knight D Gerke V Bader MF Annexin 2 promotes the formation of lipid microdomains required for calcium-regulated exocytosis of dense-core vesicles Mol Biol Cell 2005 16 3 1108 19 10.1091/mbc.E04-07-0627 15635098 PMC551477

[B146] Menke M Gerke V Steinem C Phosphatidylserine membrane domain clustering induced by annexin A2/S100A10 heterotetramer Biochemistry 2005 44 46 15296 303 10.1021/bi051585i 16285733

[B147] Zheng YH Plemenitas A Linnemann T Fackler OT Peterlin BM Nef increases infectivity of HIV via lipid rafts Curr Biol 2001 11 11 875 9 10.1016/S0960-9822(01)00237-8 11516650

[B148] Zheng YH Plemenitas A Fielding CJ Peterlin BM Nef increases the synthesis of and transports cholesterol to lipid rafts and HIV-1 progeny virions Proc Natl Acad Sci USA 2003 100 14 8460 5 10.1073/pnas.1437453100 12824470 PMC166251

[B149] Pique C Lagaudrielare-Gesbert C Delamarre L Rosenberg AR Conjeaud H Dokhelar MC Interaction of CD82 tetraspanin proteins with HTLV-1 envelope glycoproteins inhibits cell-to-cell fusion and virus transmission Virology 2000 276 2 455 65 10.1006/viro.2000.0538 11040136

[B150] Mazurov D Heidecker G Derse D HTLV-1 Gag protein associates with CD82 tetraspanin microdomains at the plasma membrane Virology 2006 346 194 204 10.1016/j.virol.2005.10.033 16325219

[B151] Mazurov D Heidecker G Derse D The inner loop of tetraspanins CD82 and CD81 mediates interactions with human T cell lymphotrophic virus type 1 Gag protein J Biol Chem 2007 282 6 3896 903 10.1074/jbc.M607322200 17166843

[B152] Ruiz-Mateos E Pelchen-Matthews A Deneka M Marsh M CD63 is not required for production of infectious human immunodeficiency virus type 1 in human macrophages J Virol 2008 82 10 4751 61 10.1128/JVI.02320-07 18321974 PMC2346747

[B153] Hemler ME Tetraspanin functions and associated microdomains Nat Rev Mol Cell Biol 2005 6 10 801 11 10.1038/nrm1736 16314869

[B154] Chichili GR Westmuckett AD Rodgers W T cell signal regulation by the actin cytoskeleton J Biol Chem 2010 285 19 14737 46 10.1074/jbc.M109.097311 20194498 PMC2863167

[B155] Strasner AB Natarajan M Doman T Key D August A Henderson AJ The Src kinase Lck facilitates assembly of HIV-1 at the plasma membrane J Immunol 2008 181 5 3706 13 18714047 10.4049/jimmunol.181.5.3706 PMC2587142

